# Reply to Dr. Orme’s “Anti-Vaccination Nuts”

**Published:** 1898-08

**Authors:** W. B. Clarke

**Affiliations:** Indianapolis, Ind.


### REPLY TO DR. ORME'S "ANTI-VACCINATION NUTS."

W. B. Clarke, M. D., Indianapolis, Ind.

Two articles by Dr. F. H. Orme, of Atlanta, headed as above, appeared in the *Medical Century* April 1st, 1897, and April 1st, 1898, whose editors refused reply. The first, a short clipping written by a New York monthly magazine space-writer, was indorsed by Dr. Orme as "a nut which the anti-vaccinationists, who are fortunately few and becoming fewer, do not attempt to crack." The meat in this nut was that "prior to 1877," especially in 1875, New York had considerable small-pox, which since then has declined, even to almost none in some years, in consequence of vaccination, the opening statement being, "the health department of New York is more than a match for small-pox." But Dr. Orme and his oracle are silent regarding the peculiarity of small-pox to come in cycles, allowing a long stretch of years of freedom to intervene, and then to step in when slipshod sanitary measures prevail. New York is strictly sanitary, and so sanitation, not fetich, controls small-pox.

Now the State of New York passed its vaccination law

April 16th, 1860, but kept on having small-pox. Its law to establish the State Board of Health was, singularly enough, not passed until May 18th, 1880 (the year after the creation of the National Board of Health), and the board's first report, for 1880, is only a little blue-backed, paper-covered affair. We will let its second report, for 1881, a more pretentious volume, crack the nut thrown down by Dr. Orme, where it says, page 5, as well as any *anti* could: "*Small-pox*—This disease is at last so well understood as an unmasked destroyer that its contagion is readily controlled and extinguished by definite rules of disinfection, and by enforcing the absolute isolation or quarantine which the local and State sanitary codes now require." Dr. Harris, the Secretary of the Board, with the usual perfunctory place-keeping style of health officials, afterwards says of vaccination, "it is as effective as it ever was," a statement no *anti* will gainsay, and one about as valuable as the one made by Dr. Orme's oracle, "Re-vaccination seems to bestow absolute protection." "Seems" is a good word when there is no small-pox around, but worthless when it comes, as will be shown when considering Dr. Orme's own article. Dr. Orme's oracle says that "the health department of New York is more than a match for small-pox," but probably takes no account of the board's report that the deaths from small-pox in 1893 in the State were 234. (How many cases does that mean?) Nor is it likely that the oracle ever read this gleam of intelligence from a New York City Board of Health report, year unnecessary: "The extraordinary prevalence of small-pox over various parts of the globe, especially in countries where vaccination has long been efficiently practiced, its occurrence in its most fatal form in persons who gave undoubted evidence of having been well vaccinated, and the remarkable susceptibility of people of all ages to re-vaccination are facts in the history of this pestilence which must lead to a reinvestigation of the whole subject of vaccination, and of its claims as a protecting agent."

Dr. Orme and his oracle do not seem to remember that Dr. Cornel said in his presidential address before the Homœopathic Medical Society of New York, "Vaccination has been a curse instead of a blessing to the race. Every physician knows that cutaneous diseases have been by it increased in frequency, severity, and variety to an alarming extent." Dr. Orme says the *antis* do not attempt to crack nuts of the kind he quotes, so perhaps he is willing to let the New York Boards of Health crack New York grown nuts, as above—it's so much easier for the *antis*.

Coming now to Dr. Orme's own article dealing with Atlanta's experience: The doctor's imagination evidently was vivified by the Jenner jubilee symposium dished up (though poorly cooked and full of error) at Atlanta at the meeting there of the American Medical Association. But this only makes the article more interesting. Let me premise by saying that, through carelessness and preconceived opinion, such is the vaccinal veraphobia pervading all "official" provaccination writing, especially "statistics" jumbled together by hospital internes, as charged and shown by Professor Pickering and Dr. Leverson, no scrap of it can be accepted as truth without thorough sifting and often by personal investigations. Dr. Orme's Atlanta figures are simply some accepted by him as true in the goodness of his heart. Let them stand as true, and they prove nothing in favor of vaccination, the testimony being only negative and indirect, not positive and direct.

He says that Atlanta last winter had 247 cases of smallpox, with only three deaths. Such a small mortality as one per cent. is so utterly at variance with the pest-house statistics of any city under similar or any conditions, that it is unreasonable on its face, especially as it is well known that pest-houses invariably intensify the disease. If passed as truth it reflects brilliantly on the treatment carried out by the physicians, and should indicate the general adoption of that treatment.

This low mortality was attributed by him to vaccination, but in the light of history I should much prefer to know the treatment. He says that the cases vaccinated in quarantine numbered 213, of which twenty-five "developed varioloid," in other words, small-pox. That is a fair proportion under the circumstances, since to vaccinate in an epidemic time predisposes toward an attack by lowering the tone of the system and its powers of resistance. Their repelling ability would have been greater if unvaccinated, and perfect if their condition were perfect. He lays stress on the item that the three deaths and confluent cases were among unvaccinated persons. That is the usual place for such cases to figure in "statistics," for the reason that the severe disease obliterates the vaccination mark, and so every doubtful case goes down "unvaccinated." Many such have recovered, and the obliterated vaccination marks reappear. Dr. Orme speaks of the vigorous measures other than vaccination resorted to by the Board of Health. Of course there was the usual isolation, *and* quarantine, *and* disinfection, *and* cremation, *and* rubber coats, *and* so forth. Why all the *ands* if vaccination is protective?

Dr. Orme says he has "never seen any serious trouble arising from the virus." Tebb testified forty consecutive days before the British Commission, recounting 842 deaths and 6,233 cases of injury, taking the deaths from official records. Dr. Orme is fortunate, or myopic. And perhaps he has had experience with only one or two of the twenty-three official viruses used during the century, each different and of different origin. His experience is so different from that of thousands of observers as to be unique. The *Virginia Medical Monthly* (1894) more correctly states the case: "The results of vaccination the present season have been such as to demand attention to the conditions which are responsible for these effects. Upon every hand physicians report an unusual number of severe cases of vaccine disease, with grave and alarming symptoms, which showed that severe septicæmia had been produced." Whereupon even the staid *Medical Record* said: "We doubt if a law compelling universal vaccination could be enacted at the present time."

Dr. Orme also says that Atlanta is safe now, being well vaccinated, and that no child can enter school without it. If, like the ostrich, the doctor and Atlanta believe that safety consists in sticking only its head under cover, then is Atlanta easily safe—that is, till small-pox comes. Give small-pox its characteristic opportunity and it will show him his error. As to her schools, Atlanta furnishes a glowing example of despicable cowardice embodied in authority in thus swinging over its children's heads the club of deprivation from education for refusal to take cutaneously a dose of septic poison, a brutal and stupid retaliatory act of no sanitary value, as the child that refuses, free to go anywhere else and play with the pupils, is healthier than the one that submits, and making the school board vicariously do the dirty work of the Board of Health. The Supreme Courts of Illinois and Wisconsin have pronounced against such unwarranted usurpation, and Indiana will soon do so. In the notable vaccination symposium to be found in the *Medico-Legal Journal*, 1896, Dr. C. A. Lindsley, Secretary of the Connecticut Board of Health, says: "I am positively opposed to compulsory vaccination. . . . The people of this country are too thoroughly imbued with a sense of personal independence to submit patiently to personal compulsion." And why should their children be so compelled? And in an editorial the *Journal* itself says that as there is no State or government supervision over virus, which is now in the hands of private parties only, for mercenary purposes only, it is doubtful if vaccination could be made compulsory unless the State assumed responsibility for its bad results.

The doctor gloated over the Gloucester epidemic of 1896 and the Montreal epidemic of 1885, attributed by him to neglect of vaccination, and he should not have done it, as he thereby displays ignorance of the conditions. In the New York *Literary Digest* of July 25th, 1896, I explained the cause of this Gloucester epidemic. Gloucester was the place where the Jenneration of disease began; in other words, whence Jenner saddled his humbug upon the world. Leicester rebelled against vaccination, and demonstrated the superiority of the "stamping out" process, so called, and a few years ago Gloucester followed her example. Nearly all were vaccinated when the epidemic broke out, and it actually ran over a month before an unvaccinated person took it (see official report in Wiltshire *Advocate*, March 19th, 1896). It was found that a sewer-trap had been left open, high tide backed sewage into the gutters, and small-pox resulted. The defect had existed about three months. Sanitary conditions were resumed, and the epidemic abated. North Gloucester, with good water and sanitation, escaped almost entirely. Dr. Orme says the anti-vaccinationists were driven to cover. What the people of Gloucester really thought of vaccination may be inferred from the fact that at the next election the anti-vaccination ticket carried every ward in the city.

Dr. Orme need not have gone back two years for an English epidemic, for last March Middlesborough reported over 500 cases. Does Dr. Orme not remember that Middlesborough not long ago received the £50 prize for being the best vaccinated town in England, over 95 per cent. being vaccinated?

Now, how about Montreal in 1885, where so many died? A few cases appeared in the slummy east end early in March, and Dr. A. M. Ross, one of the great physicians of the world, who died a year ago, in that month advised isolation and a thorough clean-up. He made personal inspection and found 10,700 rotten holes full of filth that had been years in accumulating. Streets and alleys were foul and bad smelling. His advice was disregarded, and a great cry for vaccination went up, and more than 100,000 vaccinations were made, at an expense to the citizens of at least $50,000. It was not until October that the authorities woke up and established a system of isolation, and then cleaned up, when trouble subsided. In the high-class west end, the sanitary part, there were but a few sporadic cases. A feature of this epidemic was the number of vaccinated persons admitted to hospitals, figures of which I could give. Dr. J. Emery Coderre (a name Dr. Orme will readily recognize), of the medical faculty of Victoria University, and for thirty years physician to the Hotel Dieu Hospital, compiled and reported a long list of cases (which I have), especially of children, giving name and residence, with date of previous vaccination. Dr. Orme speaks with almost "ghoulish glee" of the fright of the *antis* there, but were they any worse off than the *pros?* If any of the former deserted and sought the scarifier, they simply were not well enough grounded in their belief to have the courage of conviction, and like Asa of old, the Bible tells us of, put their faith in the physicians of the period, and were "gathered unto their fathers."

The greatest epidemic of small-pox England ever had was that of 1870-71, of which the London *Lancet*, in its issue of January 21st, 1871, says: "The cases of small-pox after vaccination amount to four-fifths of all cases," and in its issue of July 15th, 1871, "already 122,000 vaccinated persons have had the disease." In this epidemic 42,200 died in 1870-71, and several thousand the next year, and this in a country where vaccination had been compulsory and enforced since 1853. In this same epidemic Prussia lost 124,948, though compulsory re-vaccination had existed since 1835. In Bavaria 96 per cent. of the 30,742 cases were among vaccinated persons (see *Encyclopedia Britannica*, Vol. 24). The English army commission vaccinated 5,834,861 persons in India in 1884, with a staff of 4,261, and the deaths from small-pox were 333,382. Its report says: "We are thus brought face to face with the fact that notwithstanding the existence of an active vaccination service, small-pox swept over the provinces just as if there had been none. It is clear that vaccination has been incompetent to deal with the disease in its epidemic form. If sanitary work be neglected no more dependence against small-pox can be placed on vaccination. The true remedies lie elsewhere altogether, for in an epidemic year small-pox escapes from the influence of vaccination alone." Health Commissioner Reynolds, reporting on the 1894 epidemic in Chicago, in a letter to the City Council, printed in the Chicago papers of December 30th, 1894, declares "isolation is the only means of coping with an epidemic." And his annual report, two or three days later, shows that the health board made 1,000,046 free vaccinations during the year, and that over 55 per cent. of the cases of small-pox were among vaccinated persons.

Of what consequence are Dr. Orme's paltry figures compared with the above? It is such facts as these, culled from official statistics and documents, which can be largely reinforced, that have made Dr. Orme's "anti-vacs." not "few and becoming fewer," but more numerous, and induced the Royal Commission to vote unaimously in favor of no further enforcement of the compulsory vaccination laws of England.

Two nuts I should like to have Dr. Orme try to crack are (1) Why is it that in years of great small-pox epidemics in any country where full statistics are carefully kept the general mortality is actually smaller than in the years when the country was free from such epidemics, showing that small-pox may be after all a blessing? He will find the figures for this in an article by the undersigned in the *Medical Century* for February 15th, 1896. (2) Why are consumption and cancer increasing so rapidly if vaccination is not conducing thereto? The deaths from cancer in 1895 were in New York nearly double what they were ten years before, and have quadrupled in the United States and England in forty years. In Indiana our last Board of Health report (for 1896) shows only four diseases leading cancer (after leaving out lung and heart diseases). I treat this phase of the subject in a paper read before the Kentucky Homœopathic Medical Society, May 25th, 1898, which was published in the *Medical Century*, July 1st, 1898, and partially in THE HOMŒOPATHIC PHYSICIAN for June.*

Every anti-vaccinationist was once a vaccinator in practice or in principle, and has had to change by a great shock to professional and educational prejudices, modes of thought, etc. Dr. Orme, being "sot in his ways," may, like Dr. G. Kolb, of the Royal Statistical Commission of Bavaria, "believe in vaccination more strongly than in any clerical tenet or ecclesiastical dogma." Stung into an investigation, Kolb pronounced vaccination "a complete failure." This stirred up Vogt, the Professor of Statistics at Berne, who, to disprove Kolb, collected the particulars regarding 400,000 cases of small-pox, and reported, "I am compelled to admit that my belief in vaccination is absolutely destroyed." Professors Creighton and Crookshank, of London, met the same fate, as will any one else who delves in the literature. Let Dr. Orme get into the subject a little deeper than an Atlanta epidemic that sweeps away three (!) lives, and he, too, will be convinced that the *anti-vacs.*, as he facetiously calls 'em, are right. He will not be the first who, like the undersigned, "came to scoff, remain'd to pray." So great a man as Dr. Orme, whose medical word is usually law, and whose genius illumines every subject he investigates, may do a just cause great harm by longer hugging so grotesque a delusion as vaccination is now known to be, simply because of his apparent neglect to investigate the literature of the subject. The words of the great pathologist Virchow in this direction have

---

* See "The Etiology of Cancer," by Wm. B. Clarke, M. D., in THE HOMŒOPATHIC PHYSICIAN for June, 1898, page 246.

come true. Let Dr. Orme heed them: "When the public sees a doctrine which has been exhibited to them as certain, established and claiming general acceptance proved to be faulty in its very foundation, or discovered to be willful and despotic in its essential and chief tendencies, many lose faith in science."

Lest Dr. Orme or some other homœopathist might infer from the foregoing that I am opposed to all attempt at the medicinal prevention of small-pox, I will say: Why are we not, being homœopathic, satisfied with homœopathic vaccination? Cow-pox is not small-pox, nor attenuated small-pox; nor is it like small-pox. Jenner did not introduce cow-pox—in fact, in his first and second papers he savagely condemned spontaneous cow-pox. His initial vaccination, and in fact his only one when he came before the world with his scheme, was from a sore on a woman's hand, now thought by experts (beginning with Dr. Creighton, who wrote the articles "Pathology" and "Vaccination" in *Encyclopedia Britannica*), to have been syphilitic. Let Dr. Orme compare the pictorial illustrations given by Jenner with Ricord's matchless plates of syphilis (which he can do in Crookshank's great work, *History and Pathology of Vaccination*), and he will find the true resemblance. And which do Dr. Orme's sore arms—that is, his typical vaccination ulcers—resemble most, small-pox or chancre? True, his virus is bovine, and the English use humanized; but they abandoned the bovine for the reason that it caused more trouble than the human. So Jenner's virus was modified syphilis, not modified small-pox. And Boens, of Brussels, two years ago reported 2,700 cases coming under his observation wherein syphilis was caused by vaccination. Cow-pox is incapable of preventing small-pox. It is also incapable of mitigating its severity, for, as pointed out by the great Schönlein in 1832, "there were proportionately as many mild cases of small-pox before vaccination came in vogue as there have been since."

Dr. Orme's "varioloid" is simply a quibble invented in 1818 as an excuse for cases of small-pox occurring after vaccination. (As an old darkey of my acquaintance quaintly expresses it, "Call dis very ole Lord?—I calls it de very ole debbil!") Only one virus can, in the nature of things, be preventive, and this, of course, must come from small-pox—in fact, variolinum—and it must be live enough to cause some symptoms when taken internally. There is plenty of proof in homœopathic literature that it is efficacious, but it must not be expected to do even more than small-pox itself can—and many persons have had small-pox several times. Dr. Orme does not need to inject septic poison, cow-pox, into his patients to secure mitigation, but simply to follow the instructions laid down in Raue regarding the internal use of variolinum, backed up by " the unanimous testimony of ten physicians who have used it in different epidemics."

Dr. Constantine Hering, whom Dr. Orme reveres, just twenty years ago characterized vaccination as "a poisoning of the blood. . . . The progress of our school has led us to a much more certain preventive of small-pox, and also to an easy and certain and safe cure." So what need have *we* for vaccination? Speed the day when, as Raue (1881) says (*Special Pathology*, p. 1022), "vaccination will be as obsolete in medical therapeutics as inoculation, blood-letting, and kindred barbarisms of old are to-day."

---

The reply to Dr. Orme by Dr. Clarke, of Indianapolis, which is the paper herewith published, is so excellent an exposition of the subject of Vaccination that we print it entire, thereby unintentionally crowding out the usual amount of Dr. Leverson's own contributions to the subject this month.

Dr. Clarke suggested that it be abbreviated for convenience of insertion, but the editor found that this could not be done without seriously mutilating it: a procedure not to be thought of for such a valuable paper.—[Ed.]

